# Patient and Public Involvement (PPI) in evidence synthesis: how the PatMed study approached embedding audience responses into the expression of a meta-ethnography

**DOI:** 10.1186/s12874-020-0918-2

**Published:** 2020-02-10

**Authors:** S. Park, N. Khan, F. Stevenson, A. Malpass

**Affiliations:** 1 Research Department of Primary Care and Population Health (PCPH), UCL Medical School, Royal Free Campus, Rowland Hill St., London, NW3 2PY UK; 2 Faculty of Medicine and Health, University of Leeds, Leeds, LS2 9JT UK; 3 Centre for Academic Primary Care, Population Health Sciences, School of Social and Community Medicine, University of Bristol, Office 1.03b, Canynge Hall, 39, Whatley Rd, Bristol, BS8 2PS UK

**Keywords:** Meta-ethnography, Evidence synthesis, Patient and pubic involvement, User participation, Interpretation, Translation, Order constructs

## Abstract

**Background:**

Patient and public involvement (PPI) has become enshrined as an important pillar of health services empirical research, including PPI roles during stages of research development and analysis and co-design approaches. Whilst user participation has been central to qualitative evidence synthesis (QES) for decades, as seen in the Cochrane consumer network and guidelines, meta-ethnography has been slow to incorporate user participation and published examples of this occurring within meta-ethnography are sparse.

In this paper, drawing upon our own experience of conducting a meta-ethnography, we focus on what it means in practice to ‘express a synthesis’ (stage 7). We suggest the methodological importance of ‘expression’ in Noblit and Hare’s seven stage process (Noblit, GW and Hare, RD. Meta-ethnography: synthesizing qualitative studies, 1988) has been overlooked, and in particular, opportunities for PPI user participation within it.

**Methods:**

Meta-ethnography comprises a seven-stage process of evidence synthesis. Noblit and Hare describe the final 7th stage of the meta-ethnography process as ‘expression of synthesis’, emphasizing co-construction of findings with the audience. In a previous study we conducted a meta-ethnography exploring patient and student experience of medical education within primary care contexts. We subsequently presented and discussed initial meta-ethnography findings with PPI (students and patients) in focus groups and interviews. We transcribed patient and student PPI interpretations of synthesis findings. As a research team, we then translated these into our existing meta-ethnography findings.

**Results:**

We describe, with examples, the process of involving PPI in stage 7 of meta-ethnography and discuss three methodological implications of incorporating PPI within an interpretative approach to QES: (1) we reflect on the construct hierarchy of user participants’ interpretations and consider whether incorporating these additional 1st order, 2nd level constructs implies an additional logic of 3rd order 2nd level constructs of the QES team; (2) we discuss the link between PPI user participation and what Noblit and Hare may have meant by ideas of ‘expression’ and ‘audience’ as integral to stage 7; and (3) we link PPI user participation to Noblit and Hare’s underlying theory of social explanation, i.e. how expression of the synthesis is underpinned by ideas of translation and that the synthesis must be ‘translated in the audience’s (user participants) particular language’.

**Conclusions:**

The paper aims to complement recent attempts in the literature to refine and improve guidance on conducting a meta-ethnography, highlighting opportunities for PPI user participation in the processes of interpretation, translation and expression. We discuss the implications of user participation in meta-ethnography on ideas of ‘generalisability’.

## Introduction

Patient and public, or ‘user’ involvement (PPI) in research has become an important feature of developmental and analytical processes [1]. Synthesis of evidence is a well-established discipline within health services and education research, informing policy and practice decision-making. Direct user participation in evidence synthesis approaches as a whole is well established. Whilst user participation has also been central to qualitative evidence synthesis (QES) for decades, as seen in the Cochrane consumer network and in Boote et al’s [2] narrative review of case examples of public involvement in systematic reviews, some argue the evidence base for the utility of PPI/user participation to improve reviews remains weak [3]. Meta-ethnography has been slow to incorporate user participation into its methodological process, and also to reflect on the implications of user involvement practice upon the methodological underpinnings of meta-ethnography. It is, however, increasingly recognized as an important aspect of review production and dissemination.

This article reports the development of meta-ethnography – a well-defined method for synthesizing qualitative research evidence described by Noblit and Hare – to embed PPI responses in the synthesis process. We propose that this approach is one way in which to lessen the gap between evidence production and implementation of that evidence into practice.

Meta-ethnography is a seven stage process for synthesizing qualitative research evidence. This methodology is described in a seminal book by Noblit and Hare [4]. There are many examples of meta-ethnography used across healthcare and education literature, including Urietta and Noblit’s recent publication examining identity within educational literature [5]. Within the meta-ethnography method, the final 7th stage is called ‘expression’. Most existing meta-ethnographies have been expressed only to professionals and academics as research publication texts. Noblit and Hare, however, encourage researchers to express syntheses in a variety of creative ways. In this article, we explore how synthesis findings can be expressed to a participant audience and further, how their responses can be captured and interpreted by the synthesis team, to become embedded in the meta-ethnography ‘expression’. We argue that there can be value in extending the 7th stage of ‘expression of the synthesis’ to include user participants’ interpretations of the synthesis findings and reflect upon the implications of this methodological addition to ideas of (1st, 2nd and 3rd order) construct hierarchy.

### Noblit and Hare’s 7 stages of meta-ethnography

There are now over eighteen approaches available to synthesise qualitative research. Booth and Flemming et al. both provide good overviews and guidance on how to choose a QES methodological approach [6, 7]. Meta-ethnography is one increasingly popular choice used for the synthesis of qualitative research papers, first described by Noblit and Hare in 1988 ‘Meta-ethnography: Synthesizing Qualitative Studies’. Despite being one of over eighteen approaches to qualitative evidence synthesis, it remains the most widely used method [8].

Noblit and Hare’s methodology has been particularly popular in a number of subject fields including healthcare and, to a lesser extent, clinical education. A recent article by Uny, France and Noblit explores why meta-ethnography has had more impact within healthcare QES than within the field of education [9]. Their article cites our own work [10] as one of the few examples of QES that apply meta-ethnography to a (clinical) educational literature. It is this same piece of work that we base our methodological discussions upon in this article. Whilst the original review [10] included PPI members as co-applicants as part of the review team (involved in decision-making during the review), the methodological discussion in this article refers to a separately funded PatMed study which set out to explore the added value and methodological implications of PPI/user contribution to stage 7- the expression of the synthesis.

Noblit and Hare’s 1988 work outlines the identification and synthesis of qualitative research, using seven specific stages [4] (Table.1: 7 Stages of Meta-Ethnography). Noblit and Hare, acknowledge the importance of context in qualitative research, and inherent paradoxes in attempting to synthesize research whose richness and strength lies in its detail and closeness to a particular context. In their book, Noblit and Hare relate their descriptions of the meta-ethnography process, to their own research context. There is an acceptance within their writing that the operationalization of meta-ethnography methods will need to vary and adapt, according to the contextual characteristics of a particular synthesis. Adherence, however, to the principles of the seven stages of the meta-ethnography process is accepted as key to the claims that this methodology has been used to complete a particular piece of work.

**Table 1:** 7 stages of Meta-ethnography

1	Formulating the research question
2	Deciding what is relevant
3	Repeated reading of the studies, noting key concepts which are your data
4	Decide how the studies are related
5	Translation
6	Synthesizing translations
7	Expression of the synthesis

A number of factors have contributed to authors attempting to further refine and focus what actually comprises *doing* a meta-ethnography. One factor is its growing popularity and application in a number of different settings, contexts, and disciplinary research cultures. Another factor is the issue of quality control and a desire to set certain boundaries of recognizable features to help an audience (e.g. reviewer, policy-maker, practitioner) to gauge the nature and appropriateness of reported meta-ethnographic methods. While many within this field would resist an attempt to standardize the approach to this interpretative method as a specific set of applications, there is an increasing momentum to better define what is meant by engagement with each stage of this method.

The eMERGe team have developed a meta-ethnography reporting tool [11], based on the teams’ previous work exploring the problems with how each of the 7 stages of meta-ethnography has been conducted and reported [12]. We share the eMERGe team’s position that ‘expression of the synthesis’ has been hampered by the lack of clarity of reporting and/or the rigour in undertaking synthesis. The eMERGe reporting guidance for stage 7 has three recommendations. First to summarise the main interpretative findings of the translation and synthesis and compare them to the existing literature; second, to reflect upon and describe the strengths and limitations of the synthesis, including both the impact of the research team on the synthesis findings as well as reflections on the contexts and methods of the primary studies; third, include a section on recommendations for practice, policy and theory. The eMERGE team currently view stage 7, ‘Expressing the Synthesis’ to be a “reporting phase” occurring after the inclusion of PPI in generation and interpretation of findings within stages 4–6. Here, in this article, we re-position ‘expression of synthesis’ as an additional opportunity for PPI co-production. Noblit and Hare refer to ‘expression of synthesis’ as more than a ‘reporting phase’ as there are opportunities for a final interpretative phase when contemplating the audience for the synthesis expression:

“once the meta-ethnography is in draft form and the translations are tentatively completed, *a final interpretative phase begins.* The interpretative issue to be resolved in this phase involves determining the meanings of the meta-ethnography for the intended audience” (p.79)

What is striking to us about the published work by the eMERGe team and their guidance for stage seven is that it does not yet interrogate Noblit and Hare’s ideas of ‘audience’ nor ideas of what it is to ‘express’ a meta-ethnography (beyond ideas of reporting) and how both these terms (audience and expression) may have significance for how ‘translation’ is understood and utilised within meta-ethnography.

### A neglected stage?

Pre-dating the work of the eMERGe team is a review of the processes and practices involved in *doing* each stage of a meta-ethnography. Lee et al. list the ways in which different authors have categorized their work under the seven different stages [13]. The authors explore and clarify what is involved methodologically for each stage of the process, but do not attend to the seventh and final stage referred to by Noblit and Hare as ‘expressing the synthesis’. In bringing together and summarizing the various ways of doing each of the various meta-ethnographic stages, Lee is probably reflecting a more general neglect of attention to this stage of the process within the meta-ethnographic literature. More recent guides to conducting meta-ethnography also remain vague about stage seven. For example, Cahill et al. [14], reminds us that Noblit and Hare ask us to consider the audience when writing up the synthesis findings, but Cahill and colleagues do not state what this might or could entail.

By focusing on what it means in practice to ‘express a synthesis’ (stage 7), we are not setting out to disrupt the very positive developments of the eMERGe team in producing reporting standards. We are however, seeking to further critical discussion about how to explicitly relate different forms of PPI knowledge at different stages of the process (in making transparent how translations have been done at each stage, including stage 7).

We therefore discuss in this article how we have moved from using existing methods of PPI/user involvement during stages 1 to 6 - as reported in Park et al. [10] - to an innovative exploration of PPI/user contribution to stage 7 as part of the PatMed study.

We suggest that to date, published meta-ethnographies have overlooked the methodological importance of expression in Noblit and Hare’s seven stage process, and how user participation or PPI may be additionally related to Noblit and Hare’s interpretative ideas of ‘expression’. We attempt here to complement Lee’s work: exploring the practicalities and related methodological and theoretical issues involved in conducting a meta-ethnography, focusing here on the 7th stage of expressing the synthesis.

### The role of translation in stage 7: expressing the synthesis

Noblit and Hare refer to the process of ‘translation’ in their meta-ethnographic approach. While this process is commonly reported in relation to the analysis of primary data across published articles, it is rarely referred to in the meta-ethnography literature in relation to the process of ‘expressing the synthesis’. In this section, we discuss how Noblit and Hare define ‘translation’ in relation to stage 7-expression- and how this process might be developed to include user participants in the translation and expression of evidence.

The term ‘translation’ is used by Noblit and Hare to describe the process of examining ‘how the studies in question relate to each other’ in relation to stage 5 and synthesis of those translations at stage 6 [4] (page 39). What translation means in practice in stages 5 and 6 is now well documented [13–16].

However, Noblit and Hare also highlight opportunities to use translation not only during the analytical process (in relation to stages 5 and 6), but also during the expression of meta-ethnography findings to an audience. They describe how the focus of translations is for the purpose of enabling an audience to stretch and see the phenomena in terms of others’ interpretations and perspectives. We now, therefore, focus on how translation is described by Noblit and Hare as an integral part of the synthesis expression and how this might provide opportunities to embed user participation /PPI into the process of evidence synthesis.

Noblit and Hare discuss a variety of ways in which a meta-ethnographic synthesis might be presented or expressed to an audience. Drawing upon a social constructivist epistemology, Noblit and Hare explain how the expression of a synthesis is deliberately constructed with an audience in mind. As a result, the expression will vary depending upon the audience and will therefore involve the methodological practice of translation: ‘the translations must be rendered in the audience’s particular language’ [4] (page 29). We know this instinctively as researchers and there is a substantial literature dedicated to co-construction in research and feminist methodology [see for example [17–23]]. We contend that rarely has this been discussed in the literature, in relation to meta-ethnography.

In applying the concept of translation to stage 7 (expressing the synthesis), Noblit and Hare acknowledge the challenges embedded in this process, describing translation as ‘a dilemma of expressing the strange in the familiar’s language’ [4] (page 78). In particular, they promote consideration of ways in which the synthesis might be made relevant to the audience and encourage ‘qualitative researchers to construct adequate metaphoric translations and express the account in a way that is relevant to their audience.’ [4] (pages 77–78).

## Method

So far, we have outlined how Noblit and Hare emphasise the importance of translation in shaping the expression of the synthesis (stage 7) to a range of audiences, in terms of their relevance to a particular audience, and appreciation of others’ perspectives. Next, we take the idea of translation one stage further and describe how to incorporate user participant audiences into the final expression of the meta-ethnography.

We conducted a systematic review of published research literature about undergraduate medical education in the UK general practice [10]. Patient and student users were involved throughout this review in the design, analysis and writing stages via steering group membership. This review included a descriptive summary of all included papers and two in-depth syntheses. One synthesis examined quantitative papers to answer specific research questions about effectiveness of general practice placements. A second in-depth synthesis used meta-ethnographic methods to examine some of the theoretical and conceptual underpinnings of general practice placements used within the literature. We focused on student and patient perspectives reported in selected qualitative papers. We included student and patient representatives in our research team, but were keen to find ways of further including student and patient participants in the expression of our meta-ethnography-stage 7.

The review was published for a research and education audience as a report and paper before we acquired additional funding to conduct the PatMed Study. In PatMed we wanted to find ways to express the findings of the meta-ethnography to patient and student audiences, but also to develop a way in which their responses might be captured and embedded within the expression of our meta-ethnography conceptual models. We therefore extended Noblit and Hare’s seven stage process to include three additional steps in order to embed audience responses into the expression and development of our synthesis. These three additional steps retained Noblit and Hare’s principles of translation (see Table 2: PPI Additional steps (a-c) of Stage 7 of Meta-Ethnography).

**Table 2:** PPI components of Stage 7 of Meta-ethnography

Stage 7: Expression of the Synthesis	
Stage 7a:	Embedding audience responses to the synthesis
Stage 7b:	Synthesizing audience translations
Stage 7c:	Re-expression of synthesis

**Fig. 1:**
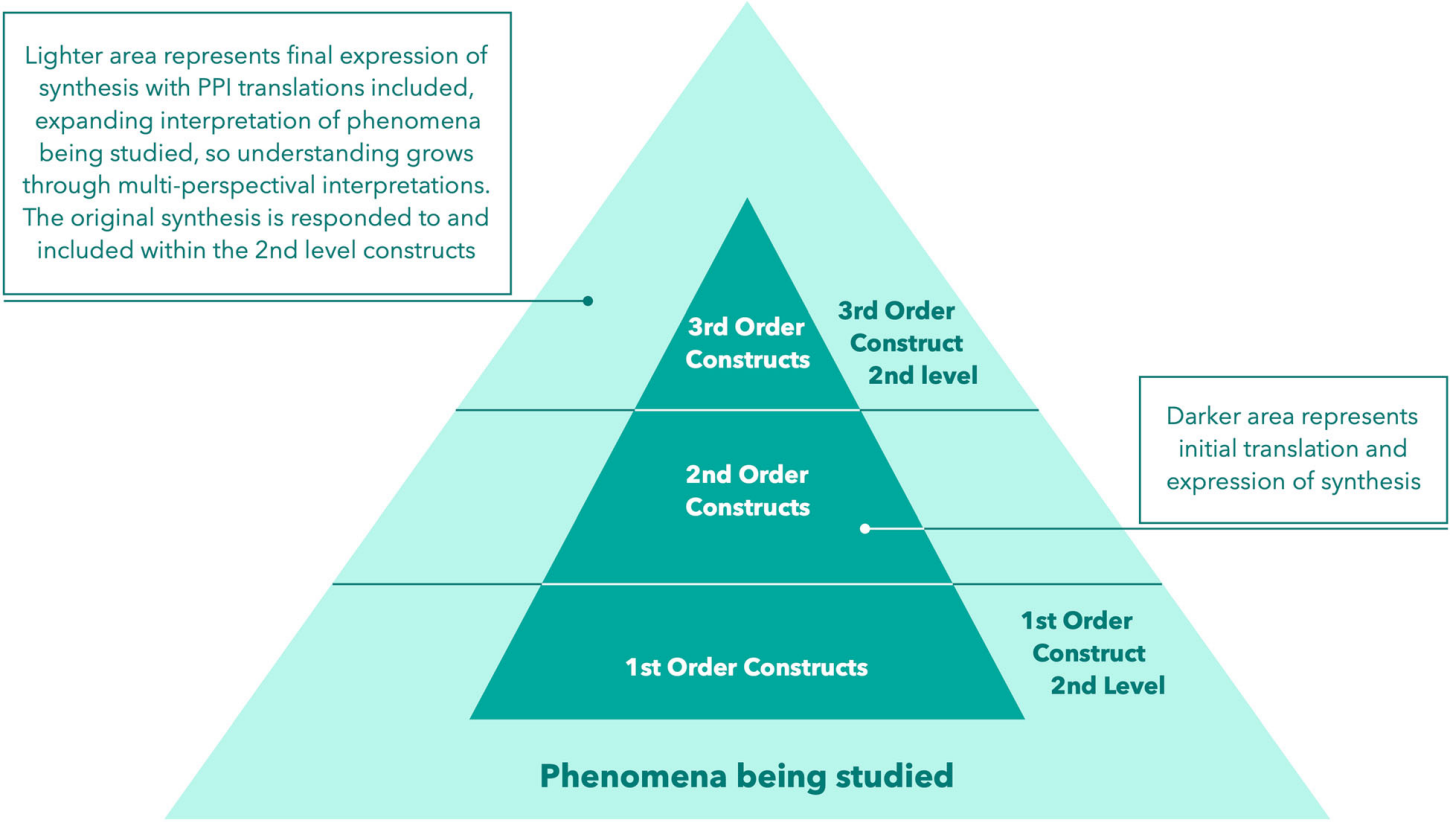
Construct Hierarchy Order Constructs in relation to levels of interpretation

We used two student participant focus groups and nine in-depth patient interviews as the ‘venue’ in which to share and express the initial synthesis findings, including presentation of two models developed during the meta-ethnography (stage 7). We then captured our audience responses to the synthesis using audio-recordings and verbatim transcription of focus group and interview interactions (stage 7a). These formed our participant interpretations of the meta-ethnography, which we have named 1st order, 2nd level interpretations (see Fig. 1: Construct Hierarchy: Order constructs in relation to levels of interpretation). Stage 7b involved the research team interpreting audience responses in relation to the meta-ethnography, and synthesizing audience translations with the existing meta-ethnography findings and models. We have named these 3rd order, 2nd level interpretations (see Fig. 1). Stage 7c then comprised an expression of (3rd order, 2nd level) interpretations, including adaptations made to meta-ethnography models as a result of 1st order, 2nd level interpretations.

### Medical student focus groups

We aimed to use familiar interaction methods for participants to promote discussion, replicating small-group teaching with our student focus groups and one-to-one consultations with our patient in-depth interviews. Student participants were recruited from two large UK-based medical schools (A and B). Recruitment emails were distributed to a final year cohort and students who had completed an intercalated BSc in Primary Care at school A, and to fourth and fifth year medical cohorts at school B. Student networks were used to advertise the study and those expressing an interest in participation were sent recruitment packs. The focus groups were conducted separately at the two schools and audio-recorded. They began with an open-ended question asking students to describe their last experience of learning in general practice. NK then showed students the two meta-ethnography models from our review: the first looking at interpersonal interactions within general practice placements, and the second exploring general practice as a distinct socio-cultural and developmental learning space. Using a student focus group guide, NK then invited comments in relation to students’ own experiences.

### Patient interviews

Patient recruitment packs (including a response sheet, information sheet and patient consent form) were distributed to GPs involved with undergraduate medical student teaching at both medical schools A and B. GPs were asked to distribute packs to patients previously involved in a teaching encounter (including an invited teaching session; student-led consultation; or impromptu medical student encounter). Patients contacted the research team directly via response sheet and were invited to interview. All interviews were conducted by NK either in person or on the phone and were audio-recorded. The interviews began with an open-ended question asking patients to describe their experiences of being involved with general practice teaching, followed by questions using an interview guide which was developed to include prompts based on the meta-ethnography and student focus groups (Appendix 2- patient interview guide). Mirroring our approach with the student focus groups, we showed patients our meta-ethnography models and invited comments based on patient experiences and views. Participants using telephone interviews, were emailed the meta-ethnography models in advance.

### Analysis

The digital audio recordings of each interview and focus group were transcribed verbatim and corrected. Focus groups and interviews were analysed by NK, then AM and SP, iteratively and coded thematically using NVivo 7 qualitative analysis software as an organising tool. Themes were labelled during initial coding, using descriptive terms grounded in participant narratives. Then, secondary descriptive categories were used to develop two separate thematic frameworks, identifying similarities and differences between interview and focus group data. During two data workshops, we discussed the thematic frameworks and how these translated across the original meta-ethnography models. We cross-tabulated patient and student views and experiences on the same issues, looking at how these views agreed, disagreed and overlapped between the two groups, and related to the meta-ethnography. We did not seek to remove aspects of the original model, but rather to refine and develop models where data provided new or different perspectives.

During analysis, we maintained on-going reflexive discussions about our own roles and experiences as researchers and how they might shape our analytical perspectives, in order to have explicit and critical discussions about counter-positions in relation to the analysis.

### Ethical approval

This study received ethical approval from the National Ethics Research Services (ref 14/LO/1550) and local Research and Development bodies. The PatMed study was funded by the NIHR School of Primary Care Research (SPCR).

## Results

**Fig. 2:**
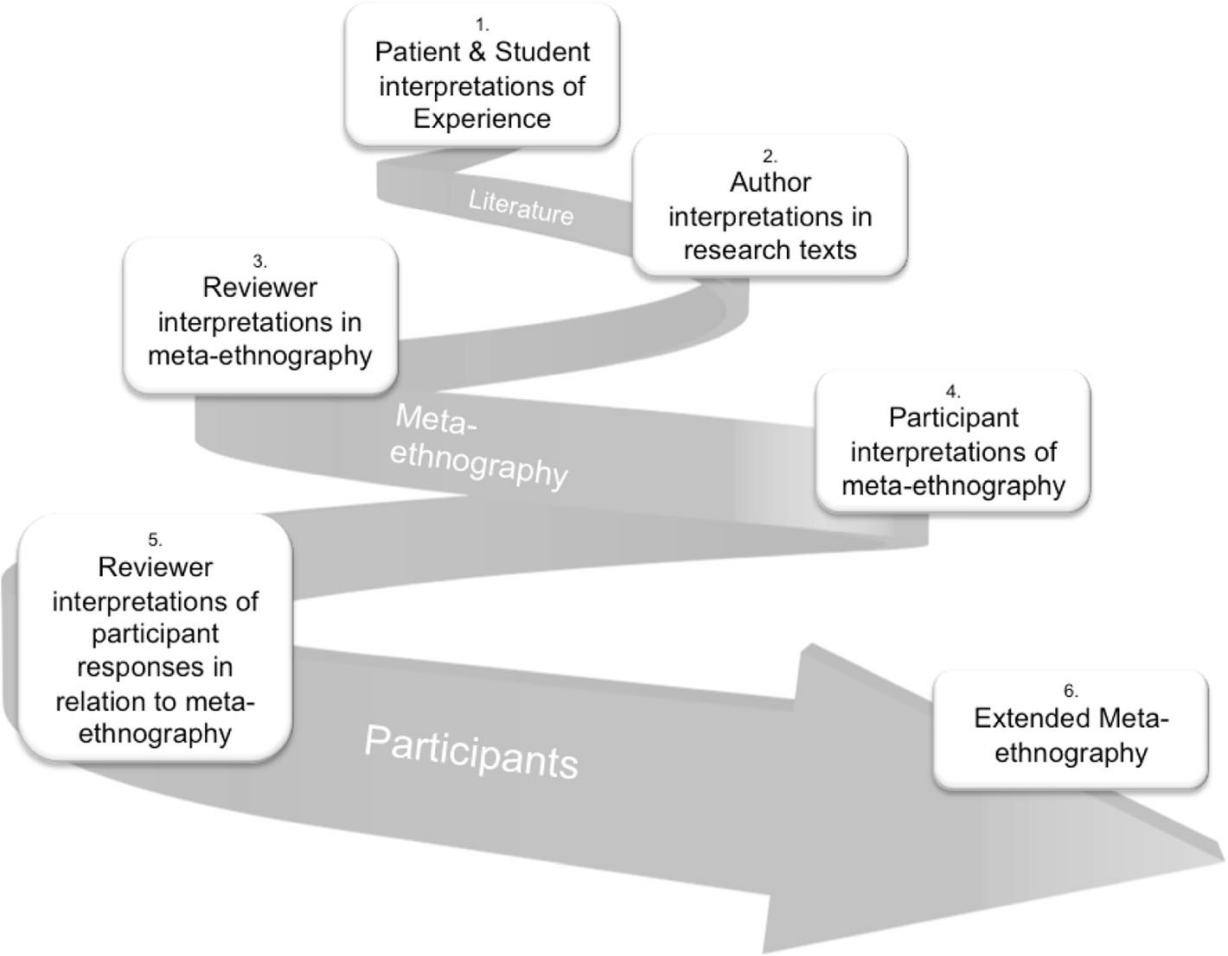
Spiral of Knowledge Construction

In this methodological paper we present our results in two sections. The first section describes, with examples, the implications of developing Noblit and Hare’s 7th stage of expression upon understandings of construct hierarchy. The second section, explores how the approach advocated here may help narrow the evidence implementation gap, illustrated with Fig. 2: Spiral of Knowledge Construction, capturing the cyclical nature of construct hierarchy we are describing.

### Interpretative steps as constructs

Some authors use the terms ‘order constructs’ to categorise the level of interpretation described within a meta-ethnography. Order construct categories were first described by Schumm et al. [24]. They report the process of meta-ethnography as movement through the relational and translational activities involved in producing first order (reported data in the research literature) and second order (author interpretations in the original studies) constructs [24]. They refer to third order constructs as the views and interpretations of the synthesis team expressed as themes and key concepts [24] (p338). We have extended this categorization to include a 2nd interpretative level of 1st and 3rd order constructs categories, in response to expression of the synthesis to participant audiences.

Figure 1: ‘Construct Hierarchy: Order constructs in relation to levels of interpretation’ summarises these new construct order categories. First order construct categories are the verbatim data reported in included papers. These are the research participants’ (medical student and patient) interpretations of their experiences that form the substance of a particular study. Second order constructs are the author’s analytical views and interpretations presented in the included papers. Tables listing first order and second order constructs were discussed with PPI members, with PPI members sharing areas of resonance and difference between first and second order constructs with their own experiences of medical education. A synthesis team then produces their own interpretations of these first and second order constructs through the process of translating these across the included studies. Again these interpretative findings were discussed with PPI members at steering group meetings. In this article, by including PPI in stages 7a, 7b and 7c (See Table 2) we add two further construct categories. Following expression of the synthesis to our participant audiences, patient and medical student audience views and responses (expressed in focus groups and interview data) become 1st order constructs, 2nd level, i.e. audience primary interpretations of the findings of the synthesis. By translating these 1st order, 2nd level constructs into one another and applying them to existing 3rd order constructs (i.e. the initial synthesis findings), we can claim to have produced 3rd order, 2nd level constructs (i.e. a re-expression of the synthesis findings that has been changed by the 1st order, 2nd level audience translations).

Our construct hierarchy is distinct from Toye’s description of 4th order constructs [25]. Toye and colleagues synthesise existing meta-ethnographies to form a mega-ethnography. For Toye, 4th order constructs are derived from the synthesis of existing meta-ethnographic 3rd order constructs.

### Narrowing the evidence translation gap

Noblit and Hare discuss how meta-ethnography syntheses can be translatable into experience [4] (page 80). In the process of engaging PPI as an audience into the expression of the synthesis, we are focusing upon the experiential knowledge of user participants about whom our meta-ethnography relates: namely students and patients. These are, of course, not the same research participants that were included and quoted in our original meta-ethnography included publications. Some qualitative research includes processes such as ‘member checking’ to make claims about the authenticity or truthfulness of findings and their alignment between their interpretations and participants’ perspectives [26]. The process we describe here is different.

This process is about new meaning-making by participants with relevant experiential knowledge, responding to the initial expression of a synthesis as an audience of experts by experience. Through facilitating a dialogue between participants and synthesis findings, we have invited our audience to participate in the process of translation and aimed to produce a further layer of interpretations of the themes and key concepts (resulting in 1st order, 2nd level constructs). Through capturing, categorizing and interpreting these in relation to the meta-ethnography findings, we have translated audience responses to our synthesis with the meta-ethnography producing another level of interpretation by the research team (3rd order, 2nd level constructs). Fig. 2: Spiral of Knowledge Construction, captures the cyclical nature of construct hierarchy we are describing here.

This process aims to embed participants’ responses to our meta-ethnography models in our now refined models and recommendations for policy and practice. In this way we have achieved what Noblit and Hare describe as the co-construction of translations: “when the synthesis is driven by some concern to inform practitioners … the audience itself may be employed to make the translations” (page 29). The practical implications of this is that we have been able to use these findings to produce information resources for (patient) participant users about participation in general practice teaching https://www.youtube.com/watch?v=YYObff2T3B0&feature=youtu.be

Table 3: Example of how embedding audience responses influenced the expression of the meta-ethnography, gives one worked example of how 1st order, 2nd level constructs changed existing 3rd order constructs into 3rd order, 2nd level constructs.

**Table 3:** Example of how embedding audience responses influenced the final expression of the meta-ethnography

Views of authors of papers included in synthesis	Views of synthesis team initial expression	PatMed participant views and ‘data responses’ to meta-ethnography models. Stage 7a	Views of synthesis team: final expression	Implications of 3rd order, 2nd level interpretations on how we understand ‘brokering’ within medical education
Stage 5 & 6	Stage 7	Stage 7a	Stage 7b	Stage 7c
2nd order 1st level	3rd order 1st level	1st order 2nd level	3rd order 2nd level	Conceptual Insights
In Primary care teaching spaces, The GP is responsible for setting up and managing the spatial arrangements and therefore controls the patient and student access to membership in the community of practice. For example, deciding where the student would sit during the teaching consultation; GP being responsible for getting (or failing to get) patient consent.	The GP as broker. This refers to how GPs set the stage of learning and guard gateway of consent. The GP is mediator and controls the nature of interactions between patient and student, as well as allowing and over-seeing membership of the community of practice.	‘Sometimes the patient is the broker, ‘cos they’re the expert on their condition, so they’re teaching the doctor a bit and they’re teaching the student a bit and they’re kind of taking control and deciding who does what and how things work.’ (S8) ‘… at my GPs, they weren’t really acting as a broker in any sense, because I would be the person who’d tell them [the patient] that they’d be seeing me first – I’d have my own room, so I’d set up a whole environment. I’d ask them if they were happy to see me before talking to the GP and explain what was going to happen.’ (S6) ‘But interestingly, on my final day … my GP tutor had been off sick, so I had to sit in with a locum who I hadn’t sat in with before, so it went back to being very passive. And you know when you’re just kind of sat observing and you don’t really get involved as much, and it felt very regressive to go back.’ (S7)	As a result of 1st order, 2nd level constructs we re-interpreted ‘The GP as broker’ with the construct: ‘Fluidity of Brokering’ Fluidity of brokering is dependent upon: (i). Patient expert knowledge (ii). Temporal student seniority (iii). Student currency (e.g. whether the student is known by the GP and therefore trusted; the gender match between student and patient)	We identified (i) the 3-dimensinoal and relational nature of brokering (ii) the flexibility between positions to ‘be broker’

## Discussion

In this paper, we have developed Noblit and Hare’s 7th stage of expression of a synthesis. In this section, we compare our example of including PPI/user participation in meta-ethnography expression with other examples in the literature before moving onto discuss how involving PPI in the expression of meta-ethnography, relates to ideas about informing practice and the ethical imperatives of generalizability.

### Involving PPI in meta-ethnography

PPI members are expected to be named reviewers as part of the synthesis team or be part of an advisory group. In our review [10] we included co-author Amanda Band, who was a member of the review steering group, and Zoya Georgieva –a student steering group member. In addition, Nada Khan – the review RA, was a medical student at the time of the review. PPI/user perspectives were, therefore, included at every stage of the review design and analysis.

We were, however, keen to find additional ways to further student and patient involvement in the expression of our meta-ethnography in stage 7-expression of the synthesis- through a second PatMed funded study. Despite guidelines from Cochrane recommending PPI members provide input into the interpretation of evidence, published examples of this occurring within meta-ethnography are sparse. One of the few examples is work by Toye et al. [15] who include PPI and stakeholders in their meta-ethnography of patients’ experience of chronic non-malignant MSK pain. Toye et al. describe setting up an advisory group that included PPI members as well as clinicians and policy makers but give little detail on how this consultation with an advisory group impacted upon their synthesis method or findings. Whilst acknowledging the valuable role PPI/user participation has in ensuring that knowledge is applicable and relevant, impacting positively upon knowledge translation, they report challenges in engaging PPI due to their variable pain levels.

A second example of a meta-ethnography team using PPI/user participation is Jamal et al. [27] (2014, 2013). This team held two face to face meetings with two separate advisory groups right at the beginning of the synthesis process to help finalise the review question and so influence search strategies and inclusion criteria decisions. They describe the consultation process with young people and professional groups as a component rather than a driver of decision making for the synthesis team. They also describe these consultations providing the team with ‘early signals’ of salient themes, suggesting then that the consultation process influenced the teams approach to data extraction and interpretation of key themes and constructs.

However, neither of these examples explore the methodological implications of including user participation on meta-ethnographic views on construct hierarchy. As far as we are aware, our work is the first to (1) reflect on the construct hierarchy of user participant interpretations and consider whether incorporating these additional 1st order, 2nd level constructs implies an additional logic of 3rd order 2nd level constructs of the QES team; (2) we are the first to discuss the link between user participation and stage 7 of meta-ethnography- expression of the synthesis; and (3) we are the first to begin to discuss in more broader terms the link between user participation and Noblit and Hare’s underlying theory of social explanation, i.e. the role of translation in audience participation if the expression of the synthesis must be ‘translated in the audience’s (user participants) particular language’.

### Purpose of syntheses – informing practice and generalizability

‘Generalisability’ is one logic used to justify the production of research literature syntheses. It is argued that by drawing together research findings, the combined ‘take homes’ might be applied in practice elsewhere in future. There are however many different ways of understanding the concept of generalizability.

Doyle argues that meta-ethnography is in itself a democratic process producing new conceptualizations of knowledge which move from the valuable stories and concepts of the particular individual cases, to the construction of a whole from those stories, which enables us to think and act differently [28] (page 339). He argues that a meta-ethnographer must be sensitive to power inequities and the subjugation of participants, minimizing the role of themselves as translator and maximizing the role of the speakers [28] (page 340). In this paper, we propose that the dominance of the researcher in the meta-ethnography process of synthesis is still present, although there is at least transparency for the reader which makes explicit the ways in which interpretations have been established.

Frost et al. [29] advocated recently that,

“We can only achieve the political imperative of knowledge synthesis (and thus real balance) *with* patients.” [29] (p.317)

Similarly, Jamal et al. [30] end their discussion of consulting with young people to inform reviews as being based upon an “ethical and political framework of participation” (p.3234). It is interesting to us that both authors (Jamal and Frost) align PPI with political imperatives.

Jamal et al. [30] identify a continuum of patient/service user participation. Consultation methods, where researchers seek the views of service users of synthesis findings or interpretations of evidence, is the most common approach adopted in systematic reviews. Consultation does not commit the synthesis team to include service user views in the final expression of the synthesis. More collaborative approaches require reviewers to work with patients and user participants on an on-going basis throughout the synthesis process as we have done within the PatMed study. The eMERGe authors to date have taken the perspective that PPIs would usually be named reviewers as part of the team or part of an additional advisory group. For us, it is important to distinguish between two types of PPI collaboration. First, PPI involvement as co-applicants and part of the review team to generate and interpret findings with certain rights to influence synthesis decisions, on one hand. Second, ‘audience-participants’ who we have worked with in stage 7, purely to explore expression of the synthesis, on the other. We see stage 7 as involving a dialogue with the intended audience, following Noblit and Hare’s description of the synthesis team members being “facilitators of a dialogue” (p.79). A dialogue between the tentative synthesis and the intended audiences is actionable, we believe, only through sharing synthesis findings with ‘audience participants’ through, for example, focus groups and/or interviews. As Noblit and Hare (1988) remind us,

“expressing an adequate synthesis and persuading an audience remain the primary goals of a meta-ethnography [and that] a meta-ethnography is complete when we understand the meaning of the synthesis to our life and the lives of others” (p.80/81)

As Harris et al. [3] argue, current research on integrated knowledge translation shows that knowledge production in any form is not solely the product of scientific expertise but a complex process of knowledge co-creation (p.211). Rarely though have ideas of knowledge co-creation been linked back to the underlying methodological stages and construct hierarchies of the synthesis process itself-as we have described here.

Harris et al. [3] use the phrase “participatory reviewing” to communicate the shift from academic expert led reviews to reviews involving “deliberative dialogue” between service users/stakeholders and academics.

What does participatory reviewing and deliberative dialogue look like in practice? Whilst not discussing meta-ethnography, Oliver et al. [31] describe in detail how consultative workshops with young people were used at different stages of the review process, the range of methods used in the workshops (such as card sorting) and the extent to which the views emerging from the consultative workshops influenced the review process and findings. The authors differentiate between using consultative workshops to check the credibility of the synthesis; to develop the implications of the review findings, (such as elements that could be included in future interventions) and identify and rank factors of importance to inform (alongside the literature) a conceptual framework for the synthesis.

Harris et al. [3] also suggest that review teams need to be aware of the ‘different types of knowledge being privileged at different stages of the review” (p.212). In our hierarchy of constructs (Fig. 1 and Table 2), we provide transparency about the different types of knowledge and orders/levels of interpretation being worked with at any one time.

### Privileging one construct over another?

The supremacy of a researcher’s interpretations have been widely critiqued in sociological research. One approach has been to include the participants directly in processes of ‘member checking’ to assess the authenticity or credibility of the researcher’s interpretations [32]. In the original meta-ethnography [10], we included patient and student representatives in our research team and steering group. What we have described here goes further. We have embedded the audience responses of patients and students into our expression of the synthesis, and included their interpretations of our meta-ethnography conceptual models into our final PatMed expression of the synthesis. For an example, see Table 3. We tried to maximize the inclusion of interaction between research papers, user participants and researchers, while making each stage of interpretation as transparent as possible to the reader (as shown in Table 2 and Figs. 1 and 2).

We did not seek to remove aspects of the original meta-ethnography models because this would have prioritized 1st order, 2nd level constructs over 1st, 2nd and 3rd order constructs. Instead we used 1st order, 2nd level and 3rd order, 2nd level constructs to refine, develop and problematize the initial (Stage 7) expression of meta-ethnography models. Nor have we attempted to go back to the original participants of included papers. That approach would conceptualise the interpretations of those individuals as fixed over time and unaffected by subsequent experience. However, we did draw upon our own experiences during the synthesis process, not only as professionals conducting a QES, but also as patients and/or medical students with our own stories of medical education in general practice settings. The authors would not have been able to embark on the meta-ethnography if they did not all share, to varying degrees, an interpretative stance on meaning-making. Our epistemological position is one of critical realism, one that views the meaning of events described in the published work in the synthesis (in this case the role of patients in medical education) as constructed, and negotiated through a process of social interaction that relates to historical and cultural systems of power. Our epistemological stance was made explicit in the first publication of our synthesis [10], drawing upon a communities of practice lens in our interpretative approach to that particular meta-ethnography. The process we have described in this article has tried, thereby, to further legitimize the involvement of user experiential knowledge in the interpretative processes of meta-ethnography. We think this is in alignment with the work of Noblit and Hare [4] and their suggestions for judging the worth and quality of a meta-ethnography:

“the worth of any synthesis is in its comprehensibility to some audience. The quality of the expression of the synthesis and its meaning (in terms of the larger human discourse, the discourse of the particular audience, and the dialogue between the two) is as dependent on the art of expression as it is on the substantive translations” (p.82). Within this article, we have set out to interrogate Noblit and Hare’s use of the terms ‘audience’ and ‘the art of expression’.

## Conclusion

Embedding user audience responses into the expression and re-expression of the synthesis, has not solved the massive issues surrounding power imbalances between researcher and the researched, inherent in most current approaches to research and evidence synthesis. However, we have shown how PPI/user participation can be methodologically embedded into the expression, interpretation and development of meta-ethnography conceptual findings. By including participant audiences in the process of research translation and the production of 1st order/ 2nd level constructs and 3rd order/ 2nd level constructs, we hope we have shifted current practice of Noblit and Hare’s stage 7 (expression of synthesis), in a direction that more closely aligns with aspirations for PPI within health services and clinical education research.

We realise that the positioning of the process of ‘audience-participation’ described in this article as being integral to stage 7 may differ from the existing eMERGe study guidance that describes stage 7 as a ‘reporting stage’. We also acknowledge that the eMERGe guidance has been developed with the input of Noblit. Our hope is that the additional perspective provided in this current article will productively contribute to debate in further enhancing and developing the inclusion of PPI in meta-ethnographic evidence syntheses, without disrupting the overall endeavour of improving reporting standards of PPI in meta-ethnography. To cite Noblit and Hare one final time, we would like to reiterate the sentiments at the end their seminal text:

“that we hope this article elicits discussion, critique and alternative proposals. We welcome this. Multiple perspectives promise us a richer and deeper understanding of our craft and our world” (p.82)

## Data Availability

The datasets used and/or analysed during the current study are available from the corresponding author on reasonable request.

## Abbreviations

eMERGe: Evidence-based meta-ethnography reporting guidance
NIHR: National Institute of Health Research
PatMed: Patient participation in undergraduate medical education in general practice study
PPI: Patient and Public Involvement
QES: Qualitative Evidence Synthesis
SPCR: School of Primary Care Research
